# Sevoflurane postconditioning protects the myocardium against ischemia/reperfusion injury via activation of the JAK2–STAT3 pathway

**DOI:** 10.7717/peerj.3196

**Published:** 2017-04-04

**Authors:** Jianjiang Wu, Jin Yu, Peng Xie, Yiliyaer Maimaitili, Jiang Wang, Long Yang, Haiping Ma, Xing Zhang, Yining Yang, Hong Zheng

**Affiliations:** 1 Department of Anesthesiology, The First Affiliated Hospital of Xinjiang Medical University, Urumqi, Xinjiang, China; 2 Department of Aerospace Medicine, Fourth Military Medical University, Xi’an, Shanxi, China; 3 Department of Cardiology, The First Affiliated Hospital of Xinjiang Medical University, Urumqi, Xinjiang, China

**Keywords:** Apoptosis, JAK2–STAT3 pathway, Reactive oxygen species, Sevoflurane postconditioning, Ischemia–reperfusion injury

## Abstract

**Background:**

Sevoflurane postconditioning (S-post) has similar cardioprotective effects as ischemic preconditioning. However, the underlying mechanism of S-post has not been fully elucidated. Janus kinase signaling transduction/transcription activator (JAK2–STAT3) plays an important role in cardioprotection. The purpose of this study was to determine whether the cardioprotective effects of S-post are associated with activation of the JAK2–STAT3 signal pathway.

**Methods:**

An adult male Sprague–Dawley (SD) rat model of myocardial ischemia/reperfusion (I/R) injury was established using the Langendorff isolated heart perfusion apparatus. At the beginning of reperfusion, 2.4% sevoflurane alone or in combination with AG490 (a JAK2 selective inhibitor) was used as a postconditioning treatment. The cardiac function indicators, myocardial infarct size, lactic dehydrogenase (LDH) release, mitochondrial ultrastructure, mitochondrial reactive oxygen species (ROS) generation rates, ATP content, protein expression of p-JAK, p-STAT3, Bcl-2 and Bax were measured.

**Results:**

Compared with the I/R group, S-post significantly increased the expression of p-JAK, p-STAT3 and Bcl-2 and reduced the protein expression of Bax, which markedly decreased the myocardial infarction areas, improved the cardiac function indicators and the mitochondrial ultrastructure, decreased the mitochondrial ROS and increased the ATP content. However, the cardioprotective effects of S-post were abolished by treatment with a JAK2 selective inhibitor (*p* < 0.05).

**Conclusion:**

This study demonstrates that the cardioprotective effects of S-post are associated with the activation of JAK2–STAT3. The mechanism may be related to an increased expression of p-JAK2 and p-STAT3 after S-post, which reduced mitochondrial ROS generation and increased mitochondrial ATP content, thereby reducing apoptosis and myocardial infarct size.

## Introduction

Ischemic heart disease is one of the major causes of death worldwide. Early restoration of hemoperfusion to the ischemic area represents the most effective method for the treatment of patients with ischemic heart disease. However, the restoration of blood flow may lead to tissue injury, termed myocardial ischemia/reperfusion (I/R) injury (Erbel & Budoff, 2012). Studies have shown that the administration of inhalation anesthetics before ischemia (anesthetic preconditioning (APC)) and immediately at the onset of reperfusion (anesthetic postconditioning) can effectively reduce myocardial I/R injury (Lemoine et al., 2016).

Compared to APC, anesthetic postconditioning is more clinically applicable and attracts more attention (Kloner & Rezkalla, 2006). Sevoflurane, a new type of inhalation anesthetic with unique physicochemical properties, including easy diffusion and translocation across the cell membrane, has been widely used for clinical anesthesia. The application of sevoflurane immediately at the onset of reperfusion (sevoflurane postconditioning (S-post)) can exert myocardial protective effects (Agarwal et al., 2014). Previous studies have shown that S-post conferred cardioprotection by attenuating mitochondrial damage, including a reduction in mitochondrial permeability transition pore (MPTP) opening (Yao et al., 2009) and an improvement in mitochondrial bioenergetics (An et al., 2005). However, the molecular mechanisms underlying S-post cardioprotective effects have not been fully elucidated.

The Janus tyrosine kinase 2 (JAK2) signal transducer and activator of transcription 3 (STAT3) pathway is an important signaling pathway, that is causatively involved in multiple physiological processes, including cell growth, differentiation, proliferation, apoptosis and inflammation (Shi et al., 2006). A previous study showed that JAK2–STAT3 is essential for ischemic postconditioning cardioprotection, and the activation of JAK2–STAT3 in ischemic postconditioning increased the expression of phosphorylated STAT3 in mitochondria, which improved mitochondrial function and eventually attenuated myocardial I/R injury (Li et al., 2016). The cardioprotective effects of S-post were diminished in hearts from diabetic rats whose cardiac STAT3 expression was decreased (Lin et al., 2016). However, whether the JAK2–STAT3 signal pathway plays a key role in S-post cardioprotection has not been studied.

In this study, we hypothesized that S-post improved mitochondrial function via the activation of the JAK2–STAT3 signal pathway, which reduced mitochondrial reactive oxygen species (ROS) generation and myocardial apoptosis, thereby attenuating myocardial I/R injury.

## Materials and Methods

### Experimental animal

This study was approved by the First Affiliated Hospital of Xinjiang Medical University, Animal Ethics Committees (IACUC-20160218-032). The experiments were performed in adherence with the National Institutes of Health Guidelines for the Use of Laboratory Animals (revised, 1996). Adult male Sprague–Dawley (SD) rats (body weight 250–300 g) were provided by the experimental animal center of the First Affiliated Hospital, Xinjiang Medical University.

### Drugs and reagents

Sevoflurane was purchased from Abbott Laboratories. Rabbit-anti-JAK2 monoclonal antibody, rabbit-anti p-JAK2 monoclonal antibody, rabbit-anti-STAT3 monoclonal antibody and rabbit-anti-p-STAT3 monoclonal antibody were purchased from Cell Signaling Technology (Danvers, MA, USA). JAK2 inhibitor AG490 was purchased from Sigma-Aldrich (St. Louis, MO, USA). Pentobarbital was purchased from Shanghai Tyrael Biological Technology Co., Ltd., Shanghai, China.

### Experimental groups

**Figure 1:**
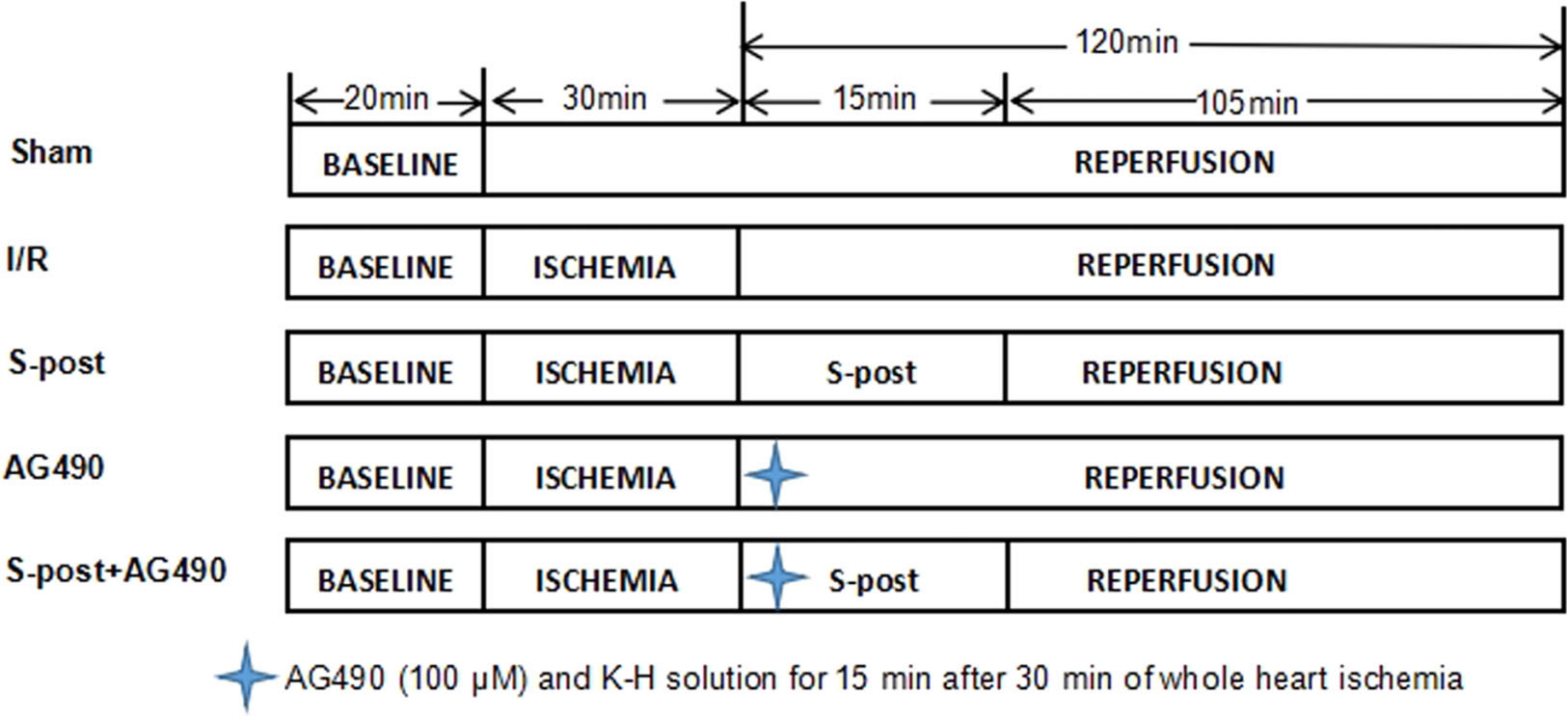
The schematic diagram of the experimental procedures. I/R, ischemic/reperfusion; S-post, sevoflurane postconditioning; AG490, JAK2 selective inhibitor.

A total of 145 rats were randomly divided into five groups (Fig. 1): (1) Sham group (Sham) (*n* = 29): The Sham group received a persistent perfusion of Krebs–Henseleit (K–H) solution for 170 min. (2) Ischemia–reperfusion group (I/R) (*n* = 29): The I/R group was equilibrated for 20 min, followed by perfusion of 4 °C St. Thomas cardioplegia; afterwards, whole heart ischemia was performed at 32 °C for 30 min, and then, the hearts were perfused with K–H solution for 120 min. (3) S-post group (*n* = 29): The S-post group was equilibrated for 20 min, followed by perfusion of 4 °C St. Thomas cardioplegia. Afterwards, whole heart ischemia was performed for 30 min at 32 °C, and then, the hearts were perfused with 1.0 MAC (minimum alveolar concentration) of sevoflurane-saturated K–H solution for 15 min, followed by continuous perfusion of K–H solution for 105 min. (4) AG490 (*n* = 29): The heart was perfused with AG490 (100 μM) and K–H solution for 15 min after 30 min of whole heart ischemia followed by continuous perfusion of K–H solution for 105 min (Barry et al., 2007; Qiao et al., 2016). (5) S-post+AG490 (*n* = 29): The heart was perfused with AG490 +1.0 MAC sevoflurane of saturated K–H solution for 15 min after 30 min of whole heart ischemia followed by continuous perfusion of K–H solution for 105 min.

In this study, we used a 1.0 MAC of sevoflurane-saturated K–H solution that was prepared by dissolving sevoflurane in K–H solution according to a procedure documented in detail by Yang et al. (2016). During the preparation, an infrared gas-analyzer (Datex-Ohmeda; GE Healthcare, Fairfield, CT, USA) and a ULT-Svi-22-07 gas detector (Division Instrument Company, Helsinki, Finland) were employed to monitor the concentration of sevoflurane in the K–H solution to ensure that the final solution reached 1.0 MAC.

### Langendorff model

The detailed methods have been described previously by our group (Yang et al., 2016). In brief, the rats were intraperitoneally injected with sodium pentobarbital and heparin. After anesthetization, the heart was rapidly removed and placed in K–H buffer pre-cooled to 4 °C to discharge all blood in the heart cavities. The heart was immobilized with a Langendorff perfusion needle and a No. 4 surgical thread. Retrograde perfusion of the aorta was performed at 37 °C using K–H solution equilibrated in 95%O_2_ to 5%CO_2_ mixed gas under 5.8 kPa perfusion pressure. To measure the pressure, an experimental pressure transducer system was connected to the pressure measuring tube, which was inserted into the left ventricle with a rubber balloon through the mitral valve after the left atrial appendage and pulmonary artery were cut open. The perfusion pressure was kept at approximately 60–70 mmHg. The left ventricular end-diastolic pressure (LVEDP) was maintained at 0–10 mmHg by adjusting the size and position of the balloon. The above steps were completed within 2 min. The inclusion criteria were a heart rate (HR) of >250 beats/min and a left ventricular developed pressure (LVDP) of >80 mmHg after the isolated heart was equilibrated for 20 min.

### Monitoring of hemodynamics

The HR (beats/min), LVEDP (mmHg), maximum rate of increase of LV pressure (+*dp*/*dt*_max_, mmHg/s) and LVDP (mmHg) at the end of reperfusion were collected with the Powerlab/8SP data collection system.

### Measurement of the myocardial infarct size

At the end of reperfusion, the heart was immediately placed in the −80 °C freezer for 7 min, and cut into 2–3 mm thick slices along the sagittal plane of the heart that were stained in 2,3,5-triphenyl-2*H*-tetrazolium chloride (TTC) solution (37 °C, 1% TTC, pH 7.4) for 25 min, and then placed in 10% formaldehyde solution for fixation overnight. The infarct area was analyzed with ImageJ software after images were taken with a digital camera.

### Determination of LDH content

As an extremely stable cytoplasmic enzyme, lactic dehydrogenase (LDH) exists in the cytoplasm of normal cells and is rapidly released outside the cell once the cytomembrane is damaged. In our study, isolated heart injury was assessed by measuring the LDH concentration in the coronary effluent. The LDH levels in the coronary effluent were determined with the same ELISA kit, following the manufacturer’s instructions.

### Myocardial ATP content measurement

The detailed methods have been described previously by Zhang et al. (2014). In brief, an ATP assay kit was used to quantify myocardial ATP based on the luciferin–luciferase reaction. The concentration of myocardial phosphocreatine was measured via reverse phase high-performance liquid chromatography. A glycogen detection kit was used to determine the concentration of glycogen in the myocardium.

### Determination of mitochondrial ROS generation

We detected the generation rate of mitochondrial ROS via fluorometric methods (Yang et al., 2016). In one reaction system, 2.9 ml of mitochondrial ROS assay medium and 0.5 mg of mitochondria were added to a 3 ml quartz cuvette. In the other reaction system, 3.3 mmol/l succinic acid was loaded as a substrate without mitochondria before the addition of 3 μl of 5 mmol/l 2′,7′-dichlorofluorescin diacetate (DCFH-DA). The two reaction system was incubated at 37 °C for 15 min, and the fluorescence intensity of the reaction system with mitochondria (sample florescence intensity) and the fluorescence intensity of the reaction system without mitochondria (basal fluorescence intensity) were then measured. The ROS generation rate was calculated by subtracting the basal fluorescence intensity from the sample florescence intensity.

### Levels of phosphorylation of p-JAK2, p-STAT3, Bcl-2 and Bax were measured via Western blot

Cardiac muscle in the risk area was stored in liquid nitrogen. A sample containing 30 μg of protein for SDS-PAGE (Invitrogen, Grand Island, NY, USA) was lysed, sealed and transferred onto a membrane. Then, it was incubated overnight with diluted p-JAK2, p-STAT3, Bcl-2 and Bax antibody solutions (4 °C, 1:1,000). The membrane was then cleaned with TBST solution. Finally, it was incubated at room temperature with HRP-tagged second antibody (1:10,000) for 1 h. The target protein bands were analyzed with the Quantity One 2.6.2 image analysis system.

### Statistical analysis

Statistical analysis was performed using the GraphPad Prism 6.0 (GraphPad Software, San Diego, CA, USA). The data are presented as the mean ± SEM. All of the data were analyzed by one-way ANOVA. Statistical analyses were performed using Student’s *t*-test for two groups and by ANOVA followed by Tukey’s post hoc test for multiple groups. *p* < 0.05 and *p* < 0.01 were considered statistically significantly.

## Results

### Cardiac function indicators

**Figure 2:**
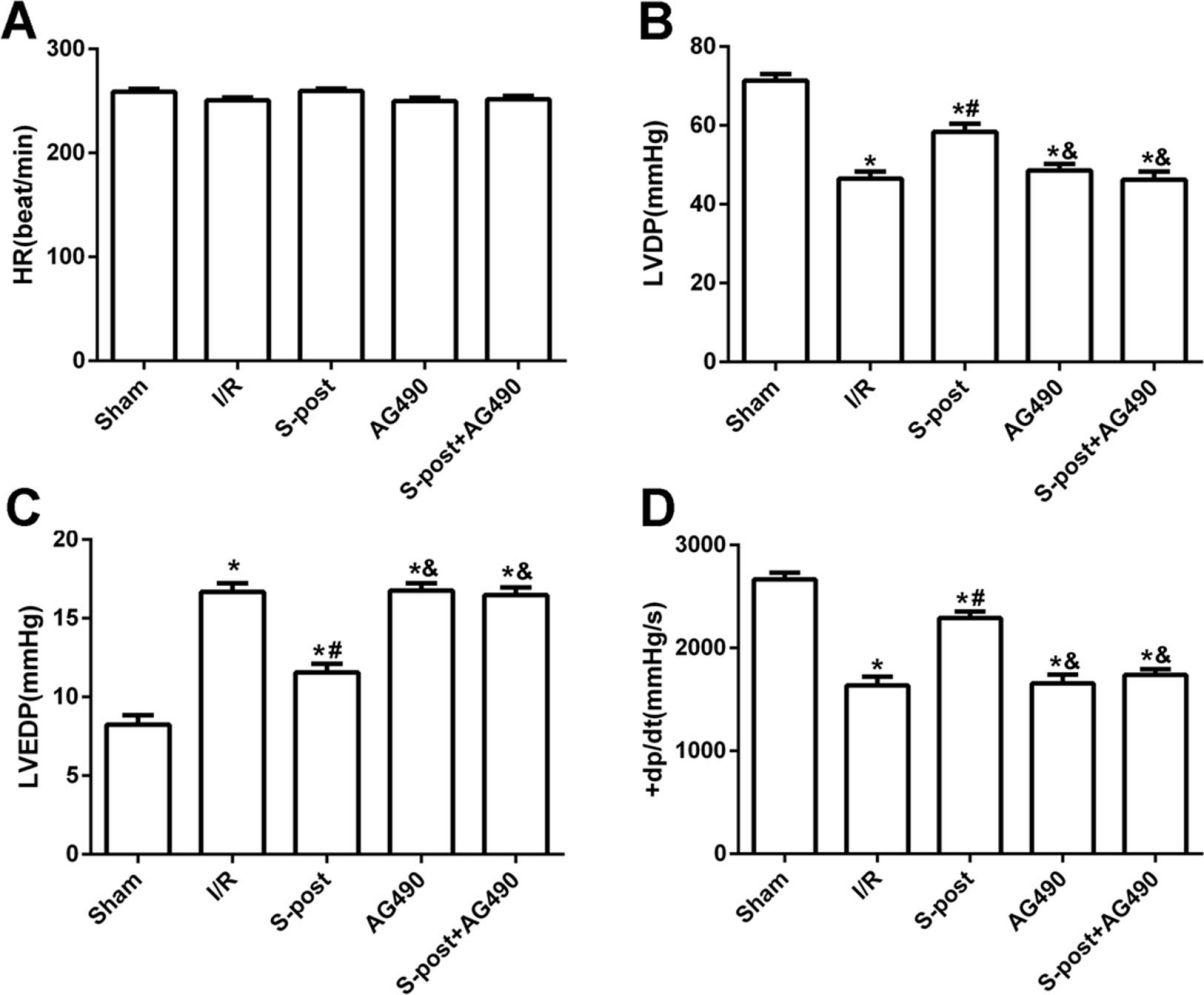
S-post improve myocardial function in vitro model. Hemodynamic changes were measured at the end of reperfusion (*n* = 12/group). (A) Heart rate (HR, beat/per min); (B) left ventricular developed pressure (LVDP, mmHg); (C) left ventricular end-diastolic pressure (LVEDP, mmHg); (D) maximum rate of increase of LV pressure (+*dp*/*dt*_max_, mmHg/s). *p < 0.05 compared with sham group, ^#^*p* < 0.05 compared with I/R group and ^&^*p* < 0.05 compared with S-post group.

Compared with the I/R group, S-post significantly increased LVDP and +*dp*/*dt*_max_ values, whereas LVEDP was significantly decreased at the end of reperfusion (*p* < 0.05). However, AG490 abolished the effects of S-post on the cardiac function indicators (AG490 and S-post+AG490 groups compared with the S-post group, *p* < 0.05, Figs. 2A–2D).

### Myocardial infarction area and LDH release

**Figure 3:**
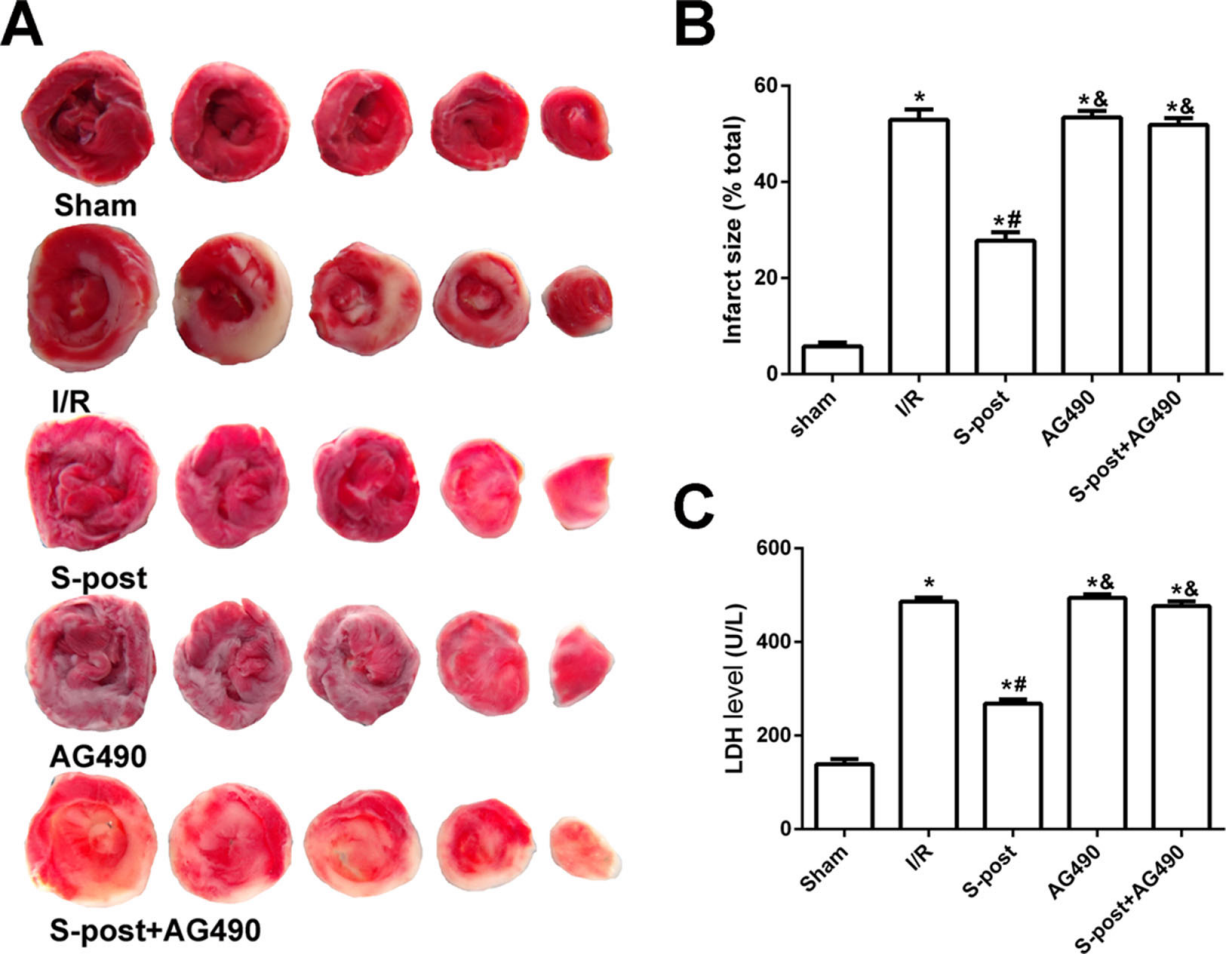
S-post reduce myocardial infarct size and LDH release (*n* = 12/group). (A) Myocardial infarct size, the infarction zone (white) and risky zone (red). (B) Myocardial infarct size (IS) expressed as the percentage of the infarct area relative to the total at-risk area after 2 h of reperfusion; (C) The LDH release level. *p < 0.05 compared with sham group, ^#^*p* < 0.05 compared with I/R group and ^&^*p* < 0.05 compared with S-post group.

Compared with the I/R group, S-post significantly decreased the myocardial infarction area and LDH release (*p* < 0.05). However, no significant differences in myocardial infarction area or LDH release were observed among the AG490, S-post+AG490 and I/R groups (*p* > 0.05, Figs. 3A–3C).

### S-post increases the levels of p-JAK2, p-STAT3 and Bcl-2, and reduces the level of Bax in the myocardium following I/R

**Figure 4:**
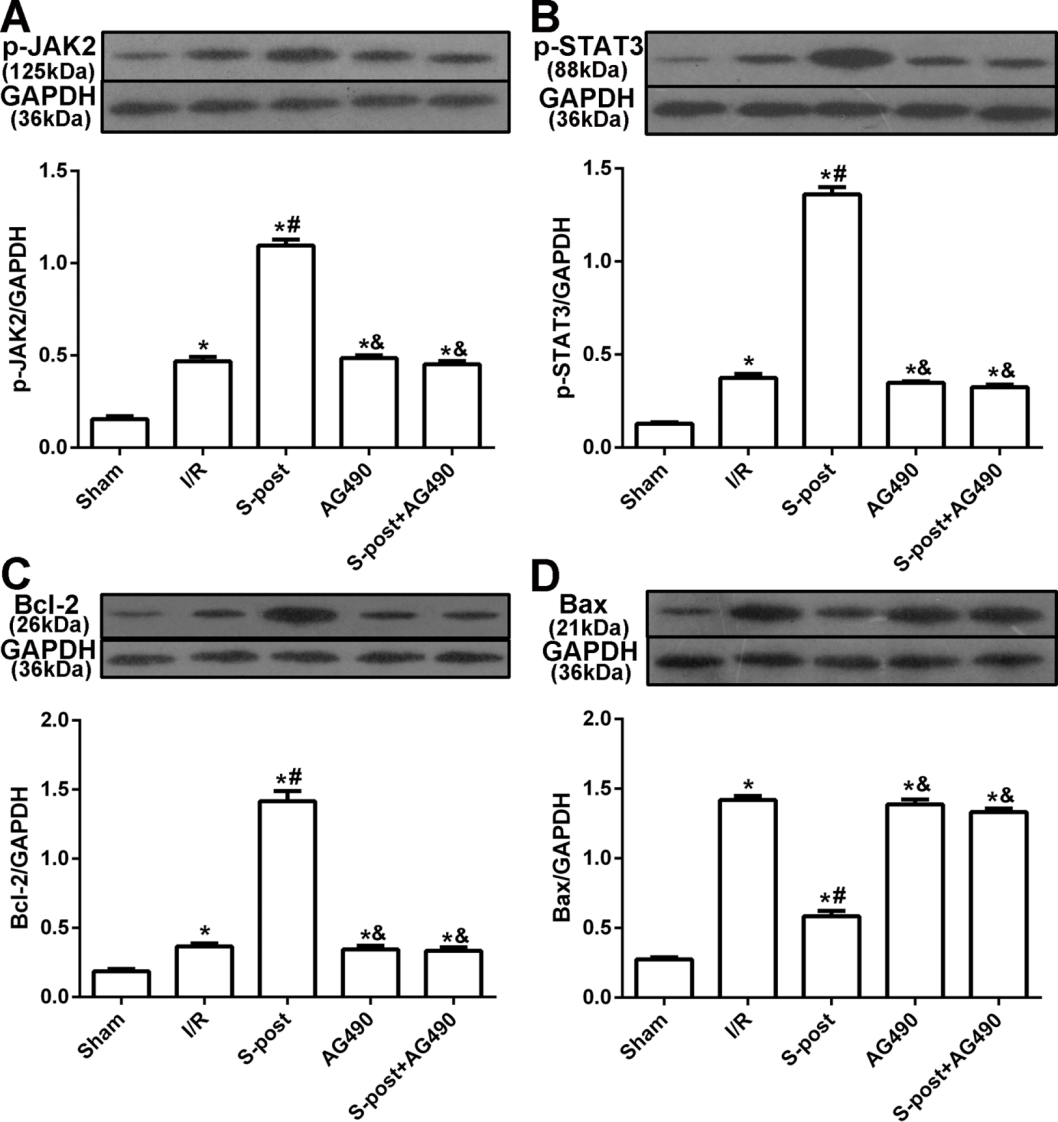
The changes in the levels of p-JAK2, p-STAT3, Bcl-2 and Bax at the end of reperfusion (*n* = 5/group). *p < 0.05 compared with sham group, ^#^*p* < 0.05 compared with I/R group and ^&^*p* < 0.05 compared with S-post group.

Compared to the I/R group, myocardial p-JAK2, p-STAT3 and Bcl-2 expression in the S-post group was significantly increased, but Bax expression was decreased (*p* < 0.05). Compared to the S-post group, myocardial p-JAK2, p-STAT3 and Bcl-2 expression in the AG490 and S-post+AG490 groups was significantly decreased, and Bax expression was increased (*p* < 0.05, Figs. 4A–4D).

### S-post alleviated mitochondrial ultrastructural damage and improved myocardial energy metabolism

**Figure 5:**
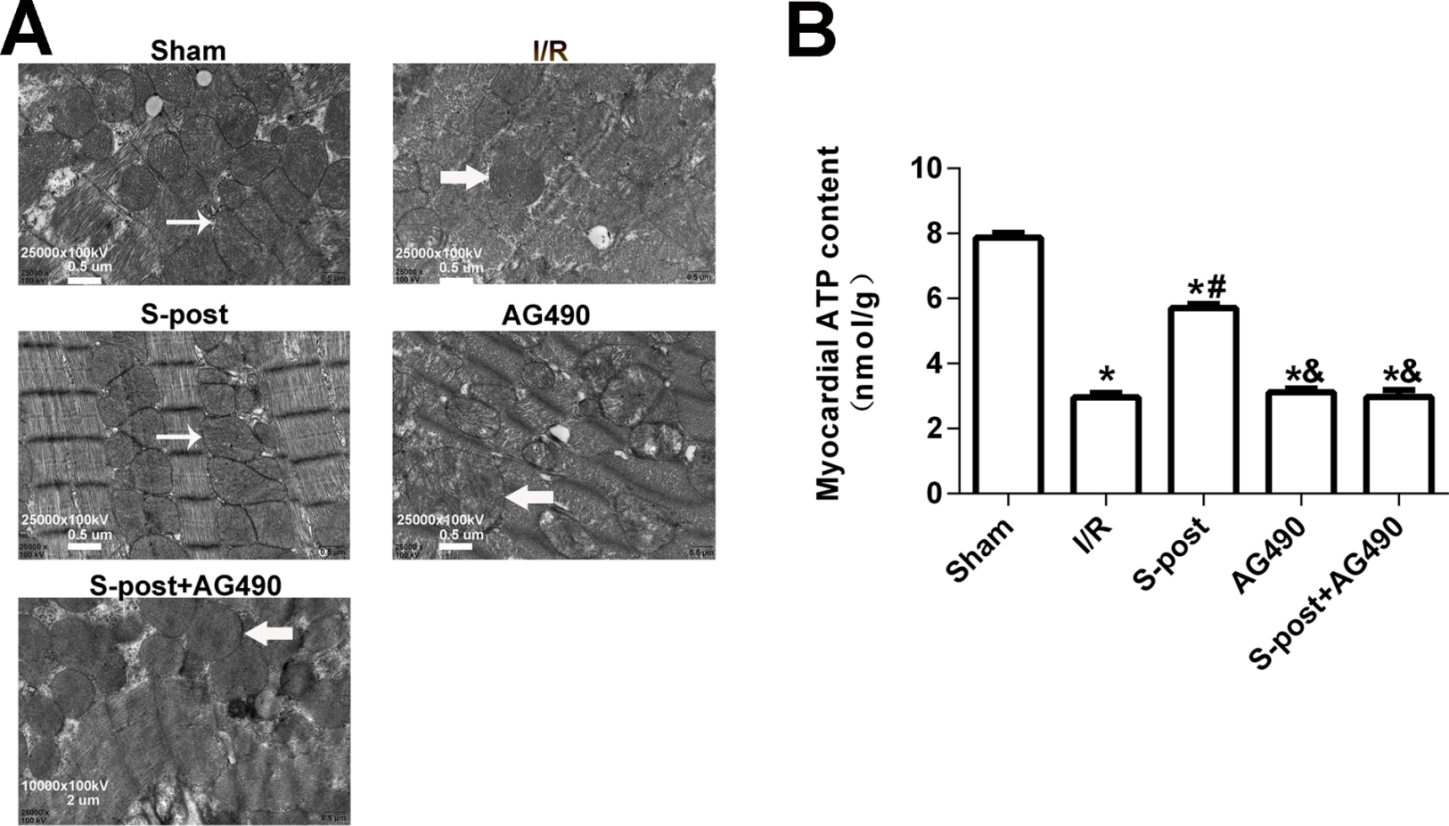
S-post alleviated mitochondrial ultrastructural damage and improved myocardial energy metabolism. (A) The changes in the mitochondrial ultrastructural (*n* = 6/group); (B) Mitochondrial ATP content (*n* = 6/group). *p < 0.05 compared with sham group, ^#^*p* < 0.05 compared with I/R group and ^&^*p* < 0.05 compared with S-post group.

In the Sham group, the mitochondrial ultrastructure was intact. The myofilaments were not dissolved, and the cristae were closely connected. In the I/R group, dissolved filaments, a dilated sarcoplasmic reticulum, swollen mitochondria, and broken cristae gaps were observed. In the S-post group, mitochondria appeared to be intact with an orderly arrangement, but slight swelling was still observed. In the S-post+AG490 and AG490 groups, the damage to the myocardial ultrastructure was similar to that in the I/R group, and no significant differences were detected between the AG490 and S-post+AG490 groups (Fig. 5A). To further confirm whether S-post improves myocardial energy metabolism, we detected myocardial ATP content via the bioluminescence method. Compared to the Sham group, the myocardial ATP content of the I/R was significantly lower. Compared to the I/R group, S-post significantly increased the ATP content (*p* < 0.05, Fig. 5B). However, there was no significant difference in myocardial ATP content among the AG490, S-post+AG490 and I/R groups (*p* > 0.05, Fig. 5B).

### Mitochondrial ROS production rates

**Figure 6:**
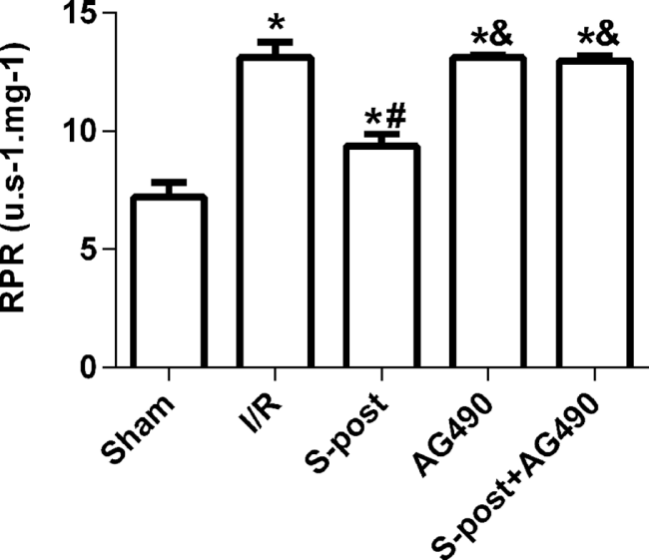
S-post reduced mitochondrial ROS production. Mitochondrial ROS production rates (RPR) at the end of reperfusion. Data are presented as the mean ± SEM (*n* = 6/group). *p < 0.05 compared with sham group, ^#^*p* < 0.05 compared with I/R group and ^&^*p* < 0.05 compared with S-post group.

The mitochondrial ROS production rates in the I/R group were significantly higher than those in the Sham group. However, S-post significantly decreased the mitochondrial ROS production rates (*p* < 0.05, Fig. 6), and the mitochondrial ROS production rates were not significantly different among the AG490, S-post+AG490 and I/R groups after the application of the JAK2 selective inhibitor; no significant differences between the AG490 and S-post+AG490 groups were detected (*p* > 0.05, Fig. 6).

## Discussion

Our study suggested that the cardioprotective effects of S-post are associated with the activation of JAK2–STAT3. The mechanism may be related to the increase in the expression of p-JAK2 and p-STAT3 after S-post, which led to reduced mitochondrial ROS generation and increased mitochondrial ATP content, thereby reducing apoptosis and myocardial infarct size.

An effective treatment of ischemic heart disease is the rapid restoration of blood flow (reperfusion) to the ischemic area. However, reperfusion may induce myocardial I/R injury. In Kersten et al. (1997) reported that volatile APC produced similar myocardial protection as ischemic preconditioning and were the first to propose the concept of APC. Studies have shown that sevoflurane preconditioning can produce myocardial protection similar to ischemic preconditioning, which can improve myocardial contractility (Obal et al., 2003) and reduce the incidence of arrhythmia (Deyhimy et al., 2007), while also improving myocardial perfusion and reducing myocardial infarct size by reducing the resistance of coronary arteries. Corresponding to APC, intervention with anesthetics during the early phase of reperfusion is known as anesthetic postconditioning (Luna-Ortiz et al., 2011). Anesthetic postconditioning can rescue myocardial ischemia and reduce infarct size to minimize reperfusion injury, and it therefore has strong practicality and operability in clinics (Zaugg et al., 2014). Studies have confirmed that S-post provides good myocardial protection and is a promising treatment method for myocardial injury (Yao et al., 2010).

Sevoflurane is a classic volatile anesthetic that has been widely used in clinical practice. During the actual application process, the MAC can be used to precisely control its inhaled and exhaled concentrations, making it simple, controllable, safe, and effective (Wang et al., 2013). Ischemic pre- and postconditioning, as well as S-post, have demonstrated significant myocardial protective effects. This is mainly achieved by elevating RISK survival signaling pathways and inhibiting Bax protein levels, thus ultimately inhibiting cardiomyocyte apoptosis (Kloner & Rezkalla, 2006; Zhang et al., 2014).

Despite significant improvements in the treatment of I/R injury, mortality and morbidity remain high in patients who have undergone cardiac surgery. Previous studies show that S-post can protect the myocardium from I/R injury (Zhang et al., 2014). Compared with other intravenous and inhalational anesthetics, sevoflurane can decrease the postoperative mortality rate (De Hert et al., 2009). Isoflurane, however, induces significantly greater neurodegeneration than an equipotent MAC of sevoflurane (Liu et al., 2016). The immediate application of sevoflurane can exert myocardial protective effects in clinical anesthesia (Huseidzinovic et al., 2007). However, the mechanism that underlies S-post cardioprotection has not been fully elucidated.

The JAK2–STAT3 signal pathway is a central component of organ protection, and it is involved in many organs such as the heart, brain, kidney and liver (Das et al., 2012; Grozovsky et al., 2015; Kim et al., 2016; Li, Li & Li, 2015; Si et al., 2014). JAK2–STAT3 signal pathways play key roles in ischemic preconditioning, particularly by protecting the myocardium at a late stage of preconditioning and by upregulating protection proteins, such as LOS and COX-2, and the proportions of anti-apoptosis proteins (Gross, Hsu & Gross, 2006). Zhao et al. (2011) demonstrated that the phosphorylation of STAT3 by JAK2 protein results in an increased expression in genes related to cell survival. Activated STAT3 is related to antioxidant effects, including the inhibition of the opening of the MPTP, anti-inflammatory effects and the increased expression of free radical scavengers (Boengler et al., 2010; Negoro et al., 2001; Schabitz et al., 2003). A previous study conducted by Kim et al. (2016) showed that S-post reduced apoptosis by upregulating the expression of p-JAK2 and p-STAT3 after transient global ischemia. However, whether the JAK2–STAT3 signal pathway plays a key role in S-post cardioprotection has not been studied. In this study, we demonstrated that S-post significantly increased the expression of p-JAK2 and p-STAT3, which markedly decreased the myocardial infarction area, improved cardiac function indicators and mitochondrial ultrastructure, regulated downstream ROS levels, suppressed myocardial cell apoptosis and the cardioprotective effects of S-post were abolished by the JAK2 selective inhibitor.

Some studies showed that myocardial apoptosis is an important aspect of myocardial I/R injury, which has been widely recognized (Huang et al., 2015; Liu et al., 2014; Sodha et al., 2008). Previous investigations indicated that activated JAK2 and STAT3 are sufficient to protect the myocardium against apoptosis (Jiang et al., 2015; Tian et al., 2011). The JAK2 selective inhibitor AG490 could abolish the cardioprotective effects induced by ischemic preconditioning (Aleshin et al., 2008). Our results showed that S-post reduced apoptosis by increasing the expression of Bcl-2 and decreasing the expression of Bax. However, the administration of AG490 reversed the cardioprotective effects of S-post on apoptosis and the JAK2–STAT3 pathway.

In addition, our latest studies have shown that mitochondria are the terminal operating apparatuses of S-post mediated cardioprotection (Yu et al., 2016a, 2016b). Mitochondria are major sources of ROS during myocardial I/R. The results of the current study suggested that the downregulation of the expression of Bcl-2 may induce the generation of ROS (Alexandre et al., 2007). To explore whether ROS participates in JAK2–STAT3 signaling induced apoptosis, we evaluated the mitochondrial ROS production rates. In this study, S-post significantly decreased the mitochondrial ROS production rates, but this beneficial effect was completely abolished by AG490. Our results indicate that S-post reduced ROS production via the activation of the JAK2–STAT3 signal pathway, thereby leading to a decrease in myocardial apoptosis. During myocardial I/R injury, preconditioning can decrease mitochondrial ROS production and inhibit the translocation of Bax to mitochondria and can inhibit apoptosis, ultimately improving cell viability (Zu et al., 2011; Zuo et al., 2015c). However, by activating myocardial survival signaling pathways, postconditioning can regulate the Bcl-2/Bax balance, maintain the morphological and functional stability of mitochondria, and promote ATP synthesis, thus ultimately inhibiting cardiomyocyte apoptosis, and achieving the same myocardial protective effects as preconditioning (Zhang et al., 2014). During the reperfusion period, a large amount of ROS is generated, causing myocardial Ca^2+^ overload, which can impair mitochondrial function, induce inflammatory mediator production, and finally result in an increase in myocardial cell apoptosis (Garciarena et al., 2011). In particular, the ROS that are released in a large amount within a few minutes after the beginning of reperfusion are considered to be a key trigger leading to myocardial ischemia–reperfusion injuries (Becker, 2004). Studies have demonstrated that S-post can prevent a high level of ROS production during the reperfusion period by triggering a lower level of ROS production (Fradorf et al., 2010), thereby playing a role in the suppression of myocardial apoptosis.

Recent studies have shown that extracellular signal-regulated kinase (ERK) and phosphatidylinositol 3-kinase/protein kinase B (PI3K/Akt) are involved in S-post-mediated myocardial protection (Chen et al., 2008). The specific inhibition of ERK1/2 and PI3K/Akt can completely eliminate the myocardial protective effects of S-post (Fang et al., 2010; Hausenloy et al., 2005). In addition, mitochondrial ATP-sensitive potassium channels (mitoK_ATP_) play an important role in myocardial I/R injury (Kowaltowski et al., 2001). It has been shown that sevoflurane provides its postconditioning effect by increasing the opening probability of mitoK_ATP_ (Kohro et al., 2001; Yao et al., 2010) and that S-post can reduce the production of mitochondrial ROS. Thus, the mechanism by which S-post exerts its myocardial protective effects may also be associated with the activation of ERK1/2 and PI3K/Akt, and the opening of mitoK_ATP_, thereby reducing the production of ROS (Yang et al., 2016; Yu et al., 2015).

Reactive oxygen species are involved in many diseases, including neurological diseases (Zuo et al., 2015b), heart failure (Zuo et al., 2015a) and hypertension (Zuo et al., 2014b). A large number of studies have shown that ischemic postconditioning can significantly reduce ROS production, thereby reducing myocardial I/R injury (Barsukevich et al., 2015; Singh et al., 2012; Zhao et al., 2003). However, ROS play a dual role in myocardial protection. In the early stage of I/R, low levels of ROS can activate a variety of protective signaling pathways, while high levels of ROS can cause oxidative stress injury in cardiomyocytes. Therefore, it is necessary to minimize mitochondrial ROS production to prevent cardiomyocyte I/R injury (He et al., 2016). Recent studies have shown that pre- and postconditioning can significantly reduce ROS production, which is associated with the activation of multiple upstream signaling pathways in mitochondria, eventually stabilizing mitochondrial function and inhibiting apoptosis (Jin et al., 2012; Penna et al., 2009; Zuo et al., 2014a, 2015c). Yu et al. (2015) and our group have found that the myocardial protection provided by S-post is associated with an inhibition of mitochondrial ROS production. In this study, we confirmed that S-post activated the JAK2–STAT3 signaling pathway, upregulated Bcl-2 protein levels, and inhibited Bax protein levels, ultimately inhibiting cardiomyocyte apoptosis and reducing myocardial infarct size.

### Limitations

There are some limitations to this study. First, we only observed the potential JAK2–STAT3 signaling pathways mechanisms in the myocardial protective function of S-post; the role of other signaling pathways should be considered in future studies. Second, this study did not involve mitochondrial morphology and function-related content, and further studies of these factors should be conducted. Finally, further experiments will need to be conducted to determine whether pre- and postconditioning have different effects on mitochondrial ROS.

## Conclusion

This study demonstrated that the cardioprotective effects of S-post are associated with the activation of JAK2–STAT3. The mechanism may be related to an increased the expression of p-JAK2 and p-STAT3 after S-post, which lead to reduced mitochondrial ROS generation and increased mitochondrial ATP content, thereby reducing apoptosis and myocardial infarct size.

## Supplemental Information

10.7717/peerj.3196/supp-1 Supplemental Information 1 Raw data for the indicators of five groups. Raw data for cardiac function indicators, myocardial infarction area, the level of LDH release, the levels of p-JAK2, p-STAT3, Bcl-2 and Bax, myocardial energy metabolism and mitochondrial ROS production rates.

10.7717/peerj.3196/supp-2 Supplemental Information 2 Protein expression. Raw data for the p-JAK2, p-STAT3, Bcl-2 and Bax.

## Additional Information and Declarations

### Data Availability

The following information was supplied regarding data availability: The raw data has been supplied as Supplemental Dataset Files.

## References

[ref-1] Agarwal B, Stowe DF, Dash RK, Bosnjak ZJ, Camara AK (2014). Mitochondrial targets for volatile anesthetics against cardiac ischemia–reperfusion injury. Frontiers in Physiology.

[ref-2] Aleshin A, Ananthakrishnan R, Li Q, Rosario R, Lu Y, Qu W, Song F, Bakr S, Szabolcs M, D’Agati V, Liu R, Homma S, Schmidt AM, Yan SF, Ramasamy R (2008). RAGE modulates myocardial injury consequent to LAD infarction via impact on JNK and STAT signaling in a murine model. AJP: Heart and Circulatory Physiology.

[ref-3] Alexandre J, Hu Y, Lu W, Pelicano H, Huang P (2007). Novel action of paclitaxel against cancer cells: bystander effect mediated by reactive oxygen species. Cancer Research.

[ref-4] An J, Camara AK, Riess ML, Rhodes SS, Varadarajan SG, Stowe DF (2005). Improved mitochondrial bioenergetics by anesthetic preconditioning during and after 2 hours of 27 degrees C ischemia in isolated hearts. Journal of Cardiovascular Pharmacology.

[ref-5] Barry SP, Townsend PA, Latchman DS, Stephanou A (2007). Role of the JAK–STAT pathway in myocardial injury. Trends in Molecular Medicine.

[ref-6] Barsukevich V, Basalay M, Sanchez J, Mrochek A, Whittle J, Ackland GL, Gourine AV, Gourine A (2015). Distinct cardioprotective mechanisms of immediate, early and delayed ischaemic postconditioning. Basic Research in Cardiology.

[ref-7] Becker LB (2004). New concepts in reactive oxygen species and cardiovascular reperfusion physiology. Cardiovascular Research.

[ref-8] Boengler K, Hilfiker-Kleiner D, Heusch G, Schulz R (2010). Inhibition of permeability transition pore opening by mitochondrial STAT3 and its role in myocardial ischemia/reperfusion. Basic Research in Cardiology.

[ref-9] Chen HT, Yang CX, Li H, Zhang CJ, Wen XJ, Zhou J, Fan YL, Huang T, Zeng YM (2008). Cardioprotection of sevoflurane postconditioning by activating extracellular signal-regulated kinase 1/2 in isolated rat hearts. Acta Pharmacologica Sinica.

[ref-10] Das A, Salloum FN, Durrant D, Ockaili R, Kukreja RC (2012). Rapamycin protects against myocardial ischemia–reperfusion injury through JAK2–STAT3 signaling pathway. Journal of Molecular and Cellular Cardiology.

[ref-11] De Hert S, Vlasselaers D, Barbe R, Ory JP, Dekegel D, Donnadonni R, Demeere JL, Mulier J, Wouters P (2009). A comparison of volatile and nonvolatile agents for cardioprotection during on-pump coronary surgery. Anaesthesia.

[ref-12] Deyhimy DI, Fleming NW, Brodkin IG, Liu H (2007). Anesthetic preconditioning combined with postconditioning offers no additional benefit over preconditioning or postconditioning alone. Anesthesia and Analgesia.

[ref-13] Erbel R, Budoff M (2012). Improvement of cardiovascular risk prediction using coronary imaging: subclinical atherosclerosis: the memory of lifetime risk factor exposure. European Heart Journal.

[ref-14] Fang NX, Yao YT, Shi CX, Li LH (2010). Attenuation of ischemia–reperfusion injury by sevoflurane postconditioning involves protein kinase B and glycogen synthase kinase 3 beta activation in isolated rat hearts. Molecular Biology Reports.

[ref-15] Fradorf J, Huhn R, Weber NC, Ebel D, Wingert N, Preckel B, Toma O, Schlack W, Hollmann MW (2010). Sevoflurane-induced preconditioning: impact of protocol and aprotinin administration on infarct size and endothelial nitric-oxide synthase phosphorylation in the rat heart in vivo. Anesthesiology.

[ref-16] Garciarena CD, Fantinelli JC, Caldiz CI, Chiappe de Cingolani G, Ennis IL, Perez NG, Cingolani HE, Mosca SM (2011). Myocardial reperfusion injury: reactive oxygen species vs. NHE-1 reactivation. Cellular Physiology and Biochemistry.

[ref-17] Gross ER, Hsu AK, Gross GJ (2006). The JAK/STAT pathway is essential for opioid-induced cardioprotection: JAK2 as a mediator of STAT3, Akt, and GSK-3 beta. AJP: Heart and Circulatory Physiology.

[ref-18] Grozovsky R, Begonja AJ, Liu K, Visner G, Hartwig JH, Falet H, Hoffmeister KM (2015). The Ashwell–Morell receptor regulates hepatic thrombopoietin production via JAK2–STAT3 signaling. Nature Medicine.

[ref-19] Hausenloy DJ, Tsang A, Mocanu MM, Yellon DM (2005). Ischemic preconditioning protects by activating prosurvival kinases at reperfusion. AJP: Heart and Circulatory Physiology.

[ref-20] He F, Li J, Liu Z, Chuang CC, Yang W, Zuo L (2016). Redox mechanism of reactive oxygen species in exercise. Frontiers in Physiology.

[ref-21] Huang CH, Lai CC, Yang AH, Chiang SC (2015). Myocardial preconditioning reduces kidney injury and apoptosis induced by myocardial ischaemia and reperfusion. European Journal of Cardio-Thoracic Surgery.

[ref-22] Huseidzinovic I, Barisin S, Bradic N, Milanovic R (2007). Early cardioprotective effect of sevoflurane on left ventricular performance during coronary artery bypass grafting on a beating heart: randomized controlled study. Croatian Medical Journal.

[ref-23] Jiang X, Guo CX, Zeng XJ, Li HH, Chen BX, Du FH (2015). A soluble receptor for advanced glycation end-products inhibits myocardial apoptosis induced by ischemia/reperfusion via the JAK2/STAT3 pathway. Apoptosis.

[ref-24] Jin C, Wu J, Watanabe M, Okada T, Iesaki T (2012). Mitochondrial K+ channels are involved in ischemic postconditioning in rat hearts. Journal of Physiological Sciences.

[ref-25] Kersten JR, Schmeling TJ, Pagel PS, Gross GJ, Warltier DC (1997). Isoflurane mimics ischemic preconditioning via activation of K(ATP) channels: reduction of myocardial infarct size with an acute memory phase. Anesthesiology.

[ref-26] Kim H-C, Kim E, Bae JI, Lee KH, Jeon Y-T, Hwang J-W, Lim Y-J, Min S-W, Park H-P (2016). Sevoflurane postconditioning reduces apoptosis by activating the JAK–STAT pathway after transient global cerebral ischemia in rats. Journal of Neurosurgical Anesthesiology.

[ref-27] Kloner RA, Rezkalla SH (2006). Preconditioning, postconditioning and their application to clinical cardiology. Cardiovascular Research.

[ref-28] Kohro S, Hogan QH, Nakae Y, Yamakage M, Bosnjak ZJ (2001). Anesthetic effects on mitochondrial ATP-sensitive K channel. Anesthesiology.

[ref-29] Kowaltowski AJ, Seetharaman S, Paucek P, Garlid KD (2001). Bioenergetic consequences of opening the ATP-sensitive K(+) channel of heart mitochondria. AJP: Heart and Circulatory Physiology.

[ref-30] Lemoine S, Tritapepe L, Hanouz JL, Puddu PE (2016). The mechanisms of cardio-protective effects of desflurane and sevoflurane at the time of reperfusion: anaesthetic post-conditioning potentially translatable to humans?. British Journal of Anaesthesia.

[ref-31] Li H, Yao W, Liu Z, Xu A, Huang Y, Ma XL, Irwin MG, Xia Z (2016). Hyperglycemia abrogates ischemic postconditioning cardioprotection by impairing AdipoR1/Caveolin-3/STAT3 signaling in diabetic rats. Diabetes.

[ref-32] Li L, Li H, Li M (2015). Curcumin protects against cerebral ischemia–reperfusion injury by activating JAK2/STAT3 signaling pathway in rats. International Journal of Clinical Experimental Medicine.

[ref-33] Lin J, Wang T, Li Y, Wang M, Li H, Irwin MG, Xia Z (2016). N-acetylcysteine restores sevoflurane postconditioning cardioprotection against myocardial ischemia–reperfusion injury in diabetic rats. Journal of Diabetes Research.

[ref-34] Liu J, Zhao Y, Yang J, Zhang X, Zhang W, Wang P (2016). Neonatal repeated exposure to isoflurane not sevoflurane in mice reversibly impaired spatial cognition at juvenile-age. Neurochemical Research.

[ref-35] Liu Y, Yang H, Song L, Li N, Han QY, Tian C, Gao E, Du J, Xia YL, Li HH (2014). AGGF1 protects from myocardial ischemia/reperfusion injury by regulating myocardial apoptosis and angiogenesis. Apoptosis.

[ref-36] Luna-Ortiz P, Torres JC, Pastelin G, Martinez-Rosas M (2011). Myocardial postconditioning: anaesthetic considerations. Archivos de Cardiología de México.

[ref-37] Negoro S, Kunisada K, Fujio Y, Funamoto M, Darville MI, Eizirik DL, Osugi T, Izumi M, Oshima Y, Nakaoka Y, Hirota H, Kishimoto T, Yamauchi-Takihara K (2001). Activation of signal transducer and activator of transcription 3 protects cardiomyocytes from hypoxia/reoxygenation-induced oxidative stress through the upregulation of manganese superoxide dismutase. Circulation.

[ref-38] Obal D, Scharbatke H, Barthel H, Preckel B, Mullenheim J, Schlack W (2003). Cardioprotection against reperfusion injury is maximal with only two minutes of sevoflurane administration in rats. Canadian Journal of Anaesthesia.

[ref-39] Penna C, Mancardi D, Rastaldo R, Pagliaro P (2009). Cardioprotection: a radical view free radicals in pre and postconditioning. Biochimica et Biophysica Acta.

[ref-40] Qiao S, Mao X, Wang Y, Lei S, Liu Y, Wang T, Wong GT, Cheung CW, Xia Z, Irwin MG (2016). Remifentanil preconditioning reduces postischemic myocardial infarction and improves left ventricular performance via activation of the Janus activated kinase-2/signal transducers and activators of transcription-3 signal pathway and subsequent inhibition of glycogen synthase kinase-3beta in rats. Critical Care Medicine.

[ref-41] Schabitz WR, Kollmar R, Schwaninger M, Juettler E, Bardutzky J, Scholzke MN, Sommer C, Schwab S (2003). Neuroprotective effect of granulocyte colony-stimulating factor after focal cerebral ischemia. Stroke.

[ref-42] Shi X, Franko B, Frantz C, Amin HM, Lai R (2006). JSI-124 (cucurbitacin I) inhibits Janus kinase-3/signal transducer and activator of transcription-3 signalling, downregulates nucleophosmin-anaplastic lymphoma kinase (ALK), and induces apoptosis in ALK-positive anaplastic large cell lymphoma cells. British Journal of Haematology.

[ref-43] Si YN, Bao HG, Xu L, Wang XL, Shen Y, Wang JS, Yang XB (2014). Dexmedetomidine protects against ischemia/reperfusion injury in rat kidney. European Review of Medical and Pharmacological Sciences.

[ref-44] Singh RB, Hryshko L, Freed D, Dhalla NS (2012). Activation of proteolytic enzymes and depression of the sarcolemmal Na+/K+-ATPase in ischemia–reperfused heart may be mediated through oxidative stress. Canadian Journal of Physiology and Pharmacology.

[ref-45] Sodha NR, Clements RT, Feng J, Liu Y, Bianchi C, Horvath EM, Szabo C, Sellke FW (2008). The effects of therapeutic sulfide on myocardial apoptosis in response to ischemia–reperfusion injury. European Journal of Cardio-Thoracic Surgery.

[ref-46] Tian Y, Zhang W, Xia D, Modi P, Liang D, Wei M (2011). Postconditioning inhibits myocardial apoptosis during prolonged reperfusion via a JAK2–STAT3–Bcl-2 pathway. Journal of Biomedical Science.

[ref-47] Wang J, Zheng H, Chen CL, Lu W, Zhang YQ (2013). Sevoflurane at 1 MAC provides optimal myocardial protection during off-pump CABG. Scandinavian Cardiovascular Journal.

[ref-48] Yang L, Xie P, Wu JJ, Yu J, Yu T, Wang HY, Wang J, Xia ZY, Zheng H (2016). Sevoflurane postconditioning improves myocardial mitochondrial respiratory function and reduces myocardial ischemia–reperfusion injury by up-regulating HIF-1. American Journal of Translational Research.

[ref-49] Yao Y, Li L, Li L, Gao C, Shi C (2009). Sevoflurane postconditioning protects chronically-infarcted rat hearts against ischemia–reperfusion injury by activation of pro-survival kinases and inhibition of mitochondrial permeability transition pore opening upon reperfusion. Biological and Pharmaceutical Bulletin.

[ref-50] Yao YT, Fang NX, Shi CX, Li LH (2010). Sevoflurane postconditioning protects isolated rat hearts against ischemia–reperfusion injury. Chinese Medical Journal.

[ref-51] Yu J, Maimaitili Y, Xie P, Wu JJ, Wang J, Yang YN, Ma HP, Zheng H (2016a). High glucose concentration abrogates sevoflurane post-conditioning cardioprotection by advancing mitochondrial fission but dynamin-related protein 1 inhibitor restores these effects. Acta Physiologica.

[ref-52] Yu J, Wu JJ, Xie P, Maimaitili Y, Wang J, Xia ZY, Gao F, Zhang X, Zheng H (2016b). Sevoflurane postconditioning attenuates cardiomyocyte hypoxia/reoxygenation injury via restoring mitochondrial morphology. PeerJ.

[ref-53] Yu P, Zhang J, Yu S, Luo Z, Hua F, Yuan L, Zhou Z, Liu Q, Du X, Chen S, Zhang L, Xu G (2015). Protective effect of sevoflurane postconditioning against cardiac ischemia/reperfusion injury via ameliorating mitochondrial impairment, oxidative stress and rescuing autophagic clearance. PLoS ONE.

[ref-54] Zaugg M, Lucchinetti E, Behmanesh S, Clanachan AS (2014). Anesthetic cardioprotection in clinical practice from proof-of-concept to clinical applications. Current Pharmaceutical Design.

[ref-55] Zhang J, Wang C, Yu S, Luo Z, Chen Y, Liu Q, Hua F, Xu G, Yu P (2014). Sevoflurane postconditioning protects rat hearts against ischemia–reperfusion injury via the activation of PI3K/AKT/mTOR signaling. Scientific Reports.

[ref-56] Zhao J, Li G, Zhang Y, Su X, Hang C (2011). The potential role of JAK2/STAT3 pathway on the anti-apoptotic effect of recombinant human erythropoietin (rhEPO) after experimental traumatic brain injury of rats. Cytokine.

[ref-57] Zhao ZQ, Corvera JS, Halkos ME, Kerendi F, Wang NP, Guyton RA, Vinten-Johansen J (2003). Inhibition of myocardial injury by ischemic postconditioning during reperfusion: comparison with ischemic preconditioning. AJP: Heart Circulatory Physiology.

[ref-58] Zu L, Zheng X, Wang B, Parajuli N, Steenbergen C, Becker LC, Cai ZP (2011). Ischemic preconditioning attenuates mitochondrial localization of PTEN induced by ischemia–reperfusion. AJP: Heart and Circulatory Physiology.

[ref-59] Zuo L, Chuang CC, Hemmelgarn BT, Best TM (2015a). Heart failure with preserved ejection fraction: defining the function of ROS and NO. Journal of Applied Physiology.

[ref-60] Zuo L, Diaz PT, Chien MT, Roberts WJ, Kishek J, Best TM, Wagner PD (2014a). PO_2_ cycling reduces diaphragm fatigue by attenuating ROS formation. PLoS ONE.

[ref-61] Zuo L, Hemmelgarn BT, Chuang CC, Best TM (2015b). The role of oxidative stress-induced epigenetic alterations in amyloid-beta production in Alzheimer’s disease. Oxidative Medicine and Cellular Longevity.

[ref-62] Zuo L, Pannell BK, Re AT, Best TM, Wagner PD (2015c). PO_2_ cycling protects diaphragm function during reoxygenation via ROS, Akt, ERK, and mitochondrial channels. American Journal of Physiology–Cell Physiology.

[ref-63] Zuo L, Rose BA, Roberts WJ, He F, Banes-Berceli AK (2014b). Molecular characterization of reactive oxygen species in systemic and pulmonary hypertension. American Journal of Hypertension.

