# Supplementary figures and images for: Sevoflurane postconditioning protects the myocardium against ischemia/reperfusion injury via activation of the JAK2–STAT3 pathway
Source: PeerJ. 2017 Apr 4;5:e3196. doi: 10.7717/peerj.3196 (PMC5382923; doi:10.7717/peerj.3196)

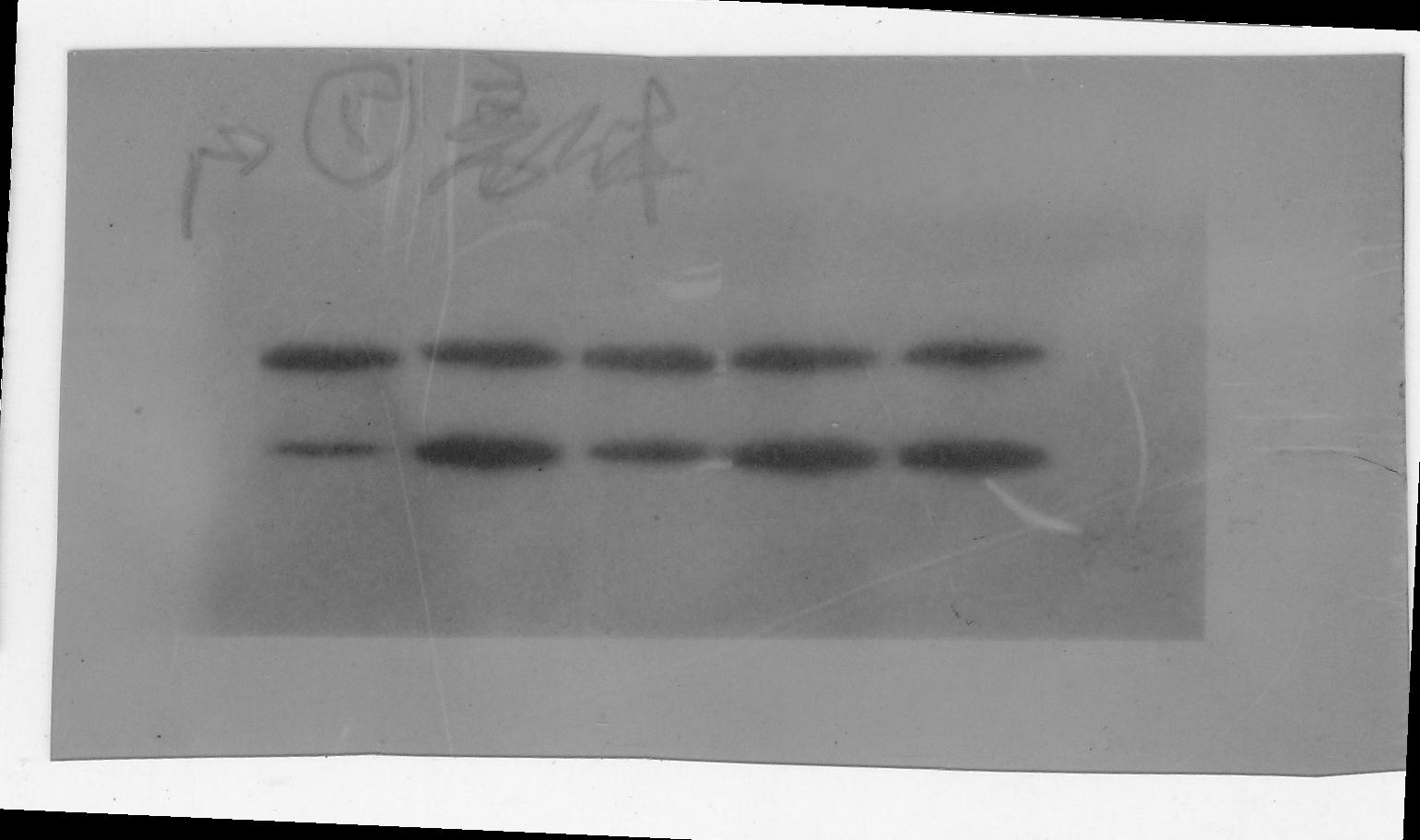

Supplement: Supplemental Information 2 — Raw data for the p-JAK2, p-STAT3, Bcl-2 and Bax. [file peerj-05-3196-s002.zip › Raw Data 2/Bax.tif]

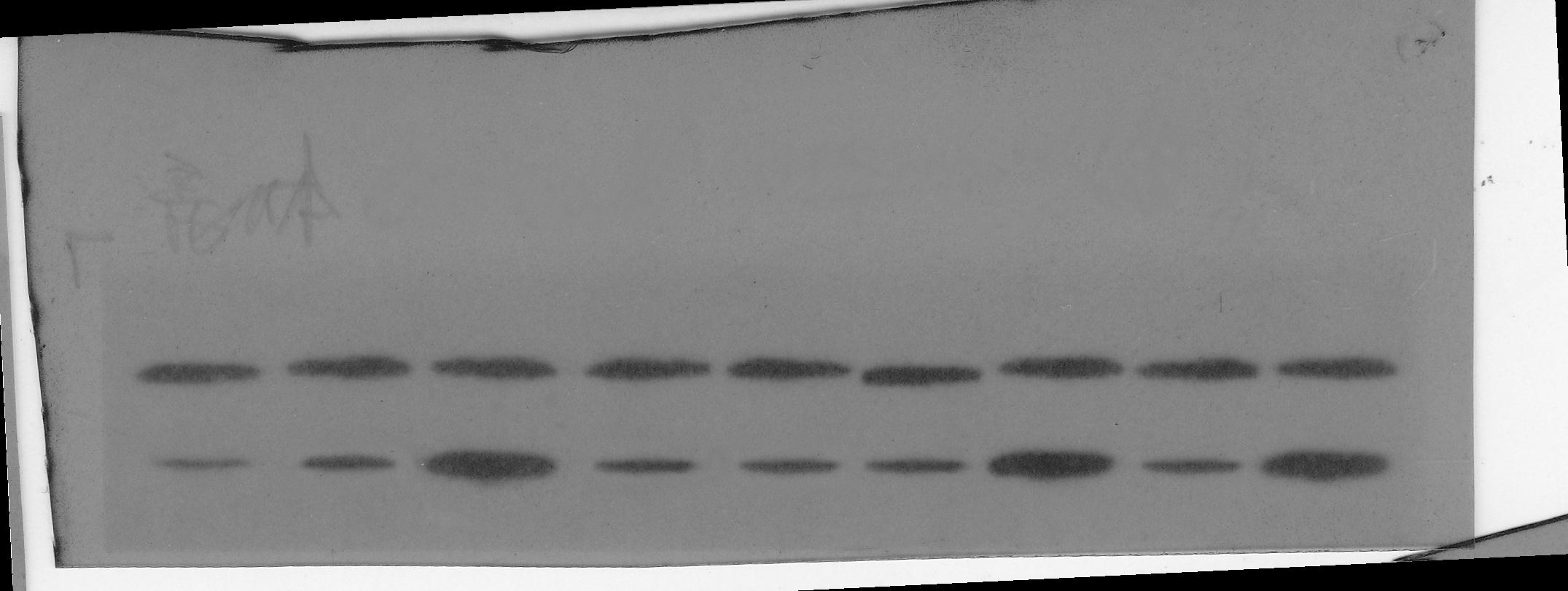

Supplement: Supplemental Information 2 — Raw data for the p-JAK2, p-STAT3, Bcl-2 and Bax. [file peerj-05-3196-s002.zip › Raw Data 2/Bcl-2(1-5 lanes).tif]

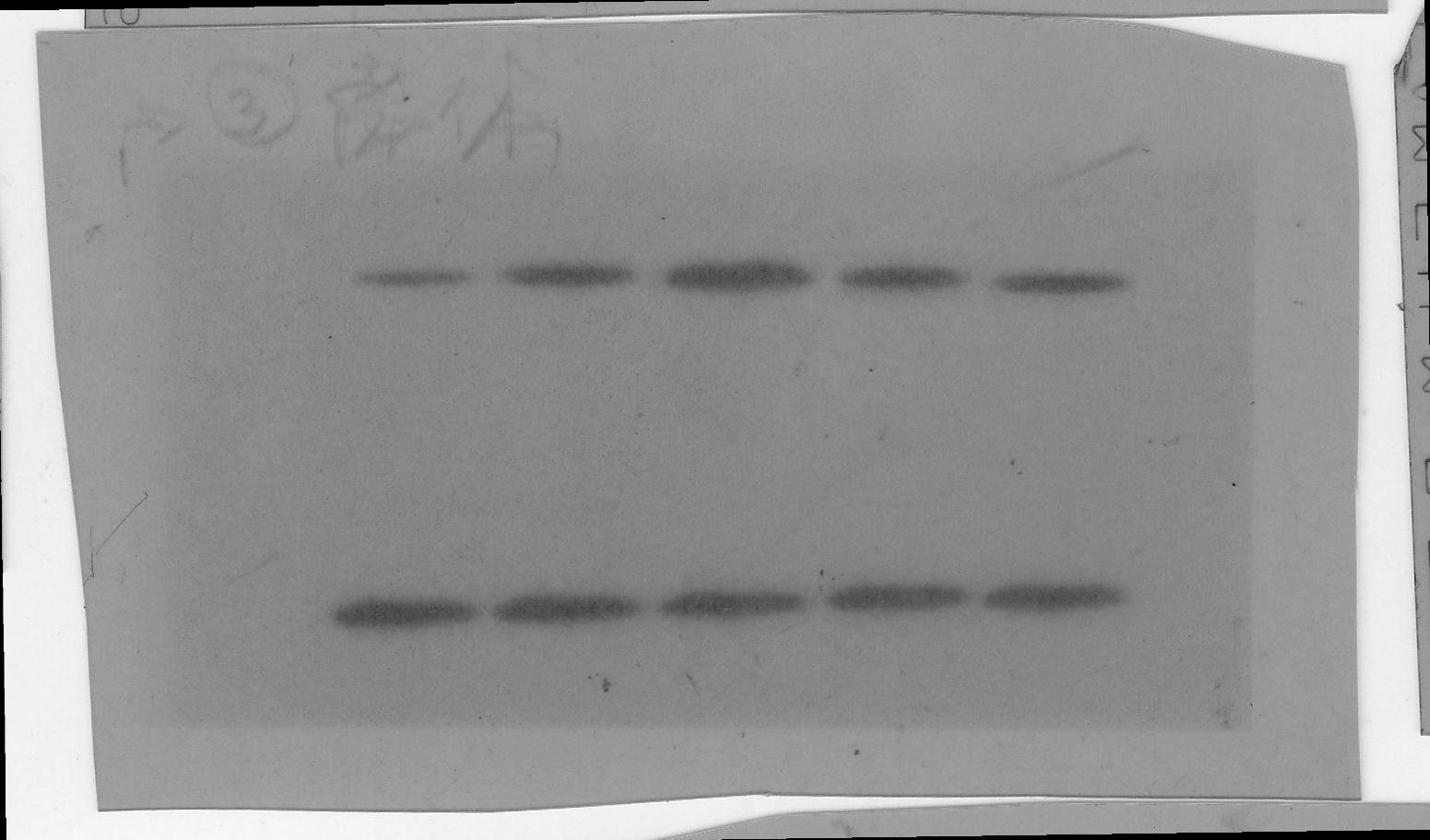

Supplement: Supplemental Information 2 — Raw data for the p-JAK2, p-STAT3, Bcl-2 and Bax. [file peerj-05-3196-s002.zip › Raw Data 2/p-JAK2.tif]

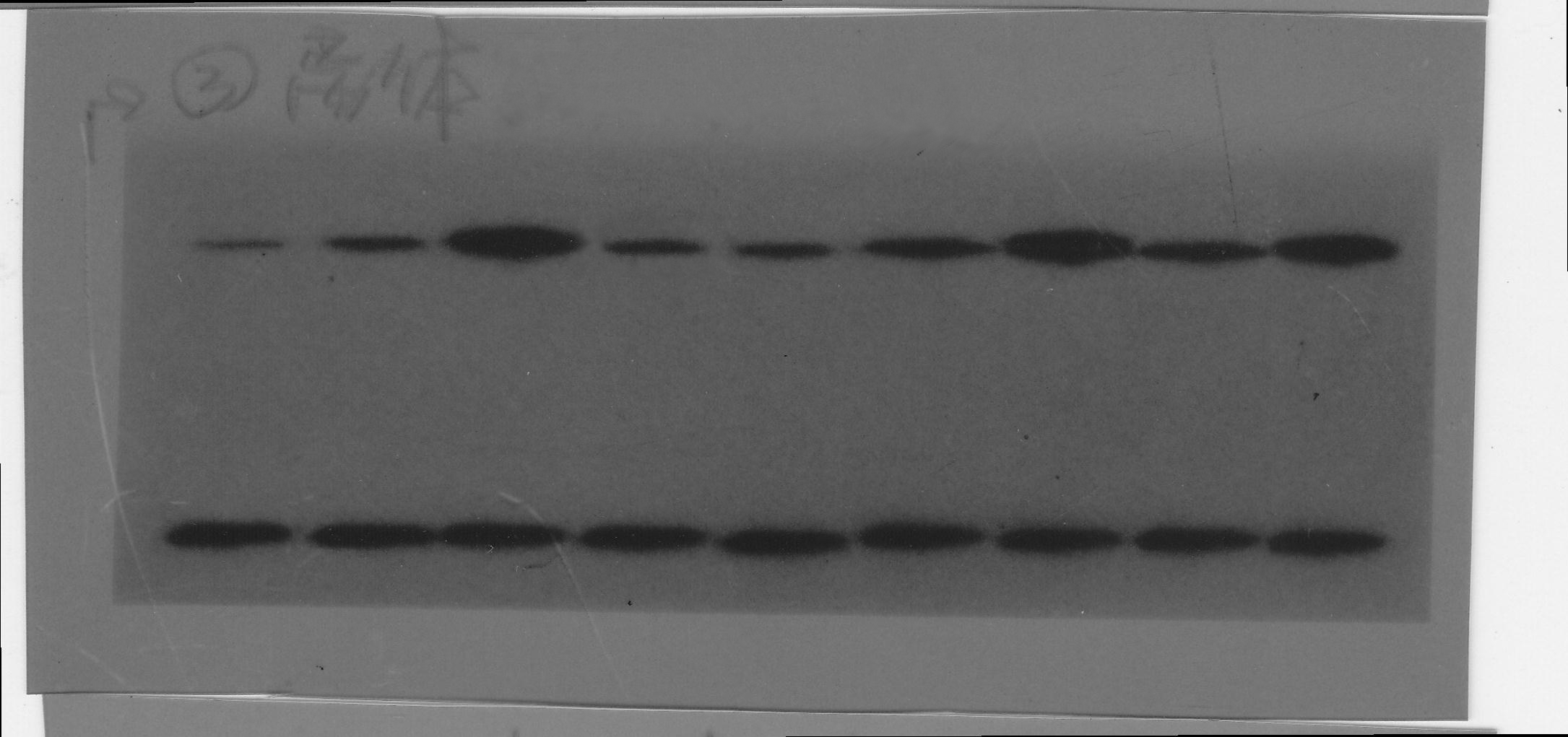

Supplement: Supplemental Information 2 — Raw data for the p-JAK2, p-STAT3, Bcl-2 and Bax. [file peerj-05-3196-s002.zip › Raw Data 2/p-STAT3(1-5 lanes).tif]
